# Phytocannabinoids in the Pharmacotherapy of Psoriasis

**DOI:** 10.3390/molecules28031192

**Published:** 2023-01-25

**Authors:** Adam Wroński, Iwona Jarocka-Karpowicz, Anna Stasiewicz, Elżbieta Skrzydlewska

**Affiliations:** 1 Dermatological Specialized Center “DERMAL” NZOZ in Bialystok, 15-453 Bialystok, Poland; 2 Department of Analytical Chemistry, Medical University of Bialystok, Mickiewicza 2D, 15-222 Bialystok, Poland

**Keywords:** phytocannabinoids, CBD, psoriasis, oxidative stress, inflammation, skin cells, blood cells

## Abstract

Phytocannabinoids are naturally occurring compounds, the main source of which is *Cannabis sativa* L. Through direct action or interaction with G protein-coupled receptors, they affect ROS and pro-inflammatory cytokines levels and modify the effectiveness of transcription factor responsible for the biosynthesis of antioxidants which lead to oxidative stress and its consequences. Due to the modification of the redox balance and inflammation, phytocannabinoids are used in the treatment of various diseases, including autoimmune dermatoses, such as atopic dermatitis and psoriasis. Psoriasis is one of the most common dermatoses, and one of unknown etiology. A disturbed redox balance with a shift towards the oxidation leads to oxidative stress, resulting in oxidative modifications, mainly of lipids and proteins, and prolonged activation of immune cells and increased generation of pro-inflammatory cytokines, resulting in chronic inflammation. Given the biological activity of phytocannabinoids, they have become the focus of research as components of pharmacotherapy for psoriasis. Beneficial effects were shown by various representatives of phytocannabinoids, but the effect of cannabidiol (CBD) on skin cells (in vitro and ex vivo) and on blood cells from patients with psoriasis vulgaris and psoriatic arthritis has been most often evaluated in recent years.

## 1. Introduction

Cannabinoids are a large group of compounds belonging to lipid mediators interacting with cannabinoid receptors (CB1 and CB2), which includes endocannabinoid, phytocannabinoids, and synthetic cannabinoids [1]. Endocannabinoids are lipid mediators, which are derivatives of polyunsaturated fatty acids, such as amides or esters (including anandamide, AEA and 2-arachidonoylglycerol, 2-AG), which are synthesized in the human organism through various metabolic pathways [2].

Synthetic cannabinoids, on the other hand, can be analogs of phytocannabinoids or endocannabinoids, as well as new compounds belonging to different groups of chemical compounds, and obtained as a result of chemical synthesis [3].

However, phytocannabinoids are a group of compounds naturally present in plants, mainly in true hemp (*Cannabis sativa* L., cannabis) [4]. The group primarily includes 21-carbon (22-carbon if present in the form of acid) terpenophenolics [4,5]. Many of these compounds are biosynthesized from olivetolic acid (OLA) and geranyl diphosphate (GPP) in trichomes, i.e., outgrowths located on almost the whole surface of the plant [6]. As a result of the reaction between OLA and GPP, cannabigerolic acid (CBGA), a phytocannabinoid precursor, is formed, whose structure includes a five-carbon side chain (mostly composed of cannabidiol, Δ^9^-tetrahydrocannabinol, and cannabichromene). A reaction between GPP and divarinic acid (DVA) is also possible, with the latter compound’s structure including a side chain containing three carbon atoms, whose product is cannabigerovarinic acid (CBGVA) [7]. Over 120 phytocannabinoids have been isolated so far, with (-)-trans-Δ^9^-tetrahydrokannabinol (Δ^9^-THC), cannabidiol (CBD), cannabigerol (CBG), and cannabichromene (CBC) receiving the most attention, and thus dubbed “The Great Four”, due to their relatively well-studied biological activity and easier synthesis compared to other phytocannabinoids [8]. Other ingredients of *Cannabis sativa* L. that belong to the group of phytocannabinoids, present in smaller quantities, also garner the interest as potential elements of pharmacotherapy. These include, among others, cannabinol (CBN), cannabinodiol (CBND), cannabielsoin (CBE), cannabicyclol (CBL), and cannabitriol (CBT) [9,10]. So far attempts have been made to use phytocannabinoids in the treatment of malaria, glaucoma, hypertension, asthma, and constipation, as well as rheumatic and labor pains—these, however, primarily concern the compound whose pharmacology is the best known, i.e., Δ^9^-tetrahydrokannabinolu (Δ^9^-THC) [11]. 

The most controversial phytocannabinoid when it comes to medical use, because of its psychotic effect, is Δ^9^-THC, which is the main component of cannabis. As a consequence, international and national organizations have established the legal status of cannabis and cannabis-derived compounds. The use of medical marijuana (in the form of a spray) is allowed in 19 countries in Europe, however smoking marijuana is prohibited even for medical purposes [12]. Canada, on the other hand, has allowed the medical/recreational use of cannabis in legally prescribed amounts [13]. In the US, only three states and one overseas dependency prohibit the use of marijuana even for medical purposes, while others allow medical and/or recreational use [14]. However, in 2020, based on a statement from the World Health Organization (WHO), the United Nations Commission on Narcotic Drugs (CND) removed cannabis and hemp resin with a low level of Δ^9^-THC from Schedule IV of substances highly harmful to public health while maintaining essential medical properties [15].

Due to the wide spectrum of phytocannabinoids’ activity in vitro, these compounds have been mainly studied so far as therapeutic agents in the treatment of neurological disorders [6,16], as well as gastrointestinal diseases [17]. Moreover, it has been shown in the last decade that phytocannabinoids might have anti-cancer properties due to its ability to modulate the proliferation and apoptosis of cancer cells [18,19].

Therefore, in recent years, the effect of phytocannabinoids on the skin and its cells has been characterized by altered metabolism, as a result of the action of physicochemical and biological factors, and as an element of the pharmacotherapy of autoimmune skin diseases of complex etiology, has also been analyzed [20]. The most common disease of this type is psoriasis, which currently affects about 4% of the population and whose incidence is constantly increasing. The currently used methods of psoriasis pharmacotherapy in many cases do not result in a cure, and topical treatment is most commonly used due to limited side effects [21,22]. Despite numerous studies on the complex pathogenesis of psoriasis, the development of pharmacotherapy still does not keep up with the needs of patients, as it does not lead to a complete cure or an inhibition of relapses.

Considering that both oxidative stress and inflammation are observed in the course of psoriasis, and that phytocannabinoids can regulate both processes directly by modulating the level/activity of endogenous compounds in skin cells involved in the regulation of these processes, as well as by direct activation of receptors cannabinoids in skin cells [23,24], there is a high probability that these compounds will create new therapeutic possibilities for autoimmune skin diseases, mainly psoriasis. Therefore, the aim of this study is to show metabolic problems in skin psoriasis and potential ways of using *Cannabis sativa* ingredients, as single compounds or in a mixture, and extracts/oils obtained from this plant. Potential pharmacotherapy of psoriasis is shown in the context of skin diseases with a similar pathogenesis.

## 2. Search Strategy

In order to prepare literature data on the search for the possibility of using phytocannabinoids in the pharmacotherapy of psoriasis, an analysis of publications on attempts to use phytocannabinoids in the effect on the skin and its cells, both in physiology and pathology caused by the disease, but also caused by physicochemical factors, was carried out. The authors analyzed the available literature, starting from in vitro studies, through ex vivo studies, to clinical trials, which until recently constituted not only a medical but also a legal problem.

### 2.1. Literature Search

In order to analyze the literature items, the PubMed database was used. The literature review covered the years from 2015 to 2022. To search articles describing the impact of phytocannabinoids on skin diseases, especially psoriasis, the following search terms were used: (skin disease, phytocannabinoids OR phytocannabinoids, psoriasis) AND (inflammation OR skin cells OR keratinocytes OR fibroblasts OR oxidative stress) OR (phytocannabinoids, skin cells OR phytocannabinoids, inflammation OR phytocannabinoids, clinical study OR cannabis, skin inflammatory disease). In order to find legal regulations relating to *Cannabis sativa*, commercial internet search engines were used.

### 2.2. Inclusion and Exclusion Criteria

During the initial analysis, articles were prioritized taking into account full-text works in English. Letters to the editor and short summaries were omitted during the search.

### 2.3. Data Extraction

The selection of articles and data analysis was performed independently by two authors. After the initial selection, related articles were imported into Zotero to exclude duplicates. In addition, references from selected works were analyzed to identify publications/documents that may have escaped the basic search. All selected links have been carefully analyzed.

All necessary information (type of phytocannabinoid, dose/concentration, method of use, effects, tested dermatoses, number of patients and others) has been collected and extensively presented in the description and summarized in tables and figures.

### 2.4. Literature Screening

During the first search of the databases, 325 articles were identified and 266 were retrieved after checking for duplicates. After the initial selection, 131 articles remained, and after reading the full texts, 108 articles were included. A literature search scheme is provided in Supplementary Material.

## 3. Classification of Phytocannabinoids

Phytocannabinoids, similarly to other complex organic chemical compounds, may be classified based on various criteria. In the case of phytocannabinoids, two main criteria are used, i.e., the chemical structure and the mechanism of action.

**Figure 1:**
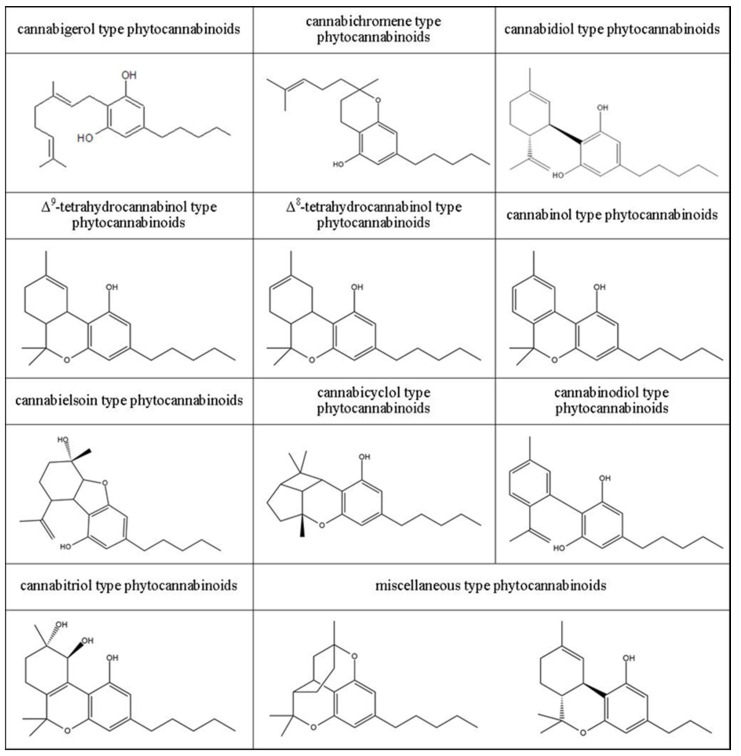
Division of phytocannabinoids by chemical structure along with chemical formulas of the main representatives of each class.

Due to the diverse chemical structure of phytocannabinoids, this group of compounds has been divided into 11 classes (Figure 1). The basic members of ten of these classes are biologically active phytocannabinoids, from which the names of individual classes and acid forms of compounds have been given [4,25]. For this reason, the following types of cannabinoids are distinguished: cannabigerol, cannabichromene, cannabidiol, Δ^9^-tetrahydrokannabinol, Δ^8^-tetrahydrokannabinol, cannabinol, cannabielsoin, cannabicyclol, cannabinodiol, and cannabitriol. Each of these classes also includes various derivatives of the main members, occurring both in the acidic and the neutral form. The final, eleventh, class of phytocannabinoids are the so-called other phytocannabinoids (miscellaneous-type phytocannabinoids), i.e., compounds with a more complex spatial structure compared to the other classes, which it would be difficult to attribute to one of the aforementioned ten classes or which could not be attributed to only one group [7,26].

The other method of classification is based on whether compounds from a given group exhibit psychoactive properties or not. It has been shown that the characteristic psychotropic effect, resulting from the activity at cannabinoid receptors CB1 located within neurons [27] is exhibited only by two out of all phytocannabinoids, i.e., (-)-trans-Δ^9^-tetrahydrokannabinol (Δ^9^-THC) and (-)-trans-Δ^8^-tetrahydrocannabinol (Δ^8^-THC) [28] and their derivatives. The effect of other compounds from the group of phytocannabinoids (e.g., cannabigerol, cannabidiol, and cannabichromene and their derivatives) on the human body results from interactions with G protein-coupled membrane receptors, including receptors CB1 and CB2, with no psychoactive effect produced [29,30]. Psychotic activity of THC and potential for addiction are the most prominent side effects observed for cannabis-derived compounds analyzed as potential pharmacotherapy ingredients [28,31].

## 4. Biological Activity of Phytocannabinoids

The biological activity of phytocannabinoids is mainly based on reacting with G protein-coupled membrane receptors, including cannabinoid, opioid, and serotonin receptors, as well as nuclear receptors, ligand gated ion channel receptors, or transient receptor potential (TRP) channels [2,30,32]. Individual phytocannabinoids show extremely varied affinity to G protein-coupled membrane receptors, including cannabinoid receptors (CB1 and CB2) (Table 1) [33]. Activation of CB1 receptors is known to cause intensified ROS and TNFα generation, which is conducive to the emergence of oxidative stress and to the triggering of cytokine storms, whereas activation of CB2 receptors is conducive to reduced ROS and TNFα generation, thus intensifying the body’s antioxidant and anti-inflammatory responses, which also takes place through immune cell regulation [33,34,35]. Moreover, the action of phytocannabinoids on cannabinoid receptors inhibits the activity of adenylyl cyclase and voltage gated calcium channels, as well as of kinase and potassium channels, including mitogen-activated protein kinase (MAPK) and phosphatidylinositol 3-kinase (PI3K)/AKT [36], with the PI3K/AKT/mTOR pathway being one of those essential for the synthesis of proteins and the induction of other intercellular pathways such as the MAPK pathway, which plays an important role in the regulation of cell survival, proliferation, and apoptosis [37].

Furthermore, in the last few years, molecular targets have been identified for some phytocannabinoids, apart from the classic endocannabinoid system. It has been shown that the compounds modulate the activity of other receptors, including peroxisome proliferator activated receptors (PPARs) [40], and interact with delta, kappa, mu opioid receptors (DOR, KOR and MOR respectively) and serotonin (5-HT_1A_) receptors, as well as with ligand gated ion channel receptors, or transient receptor potential (TRP) channels [30], thus modulating inflammation and the redox balance of the body. Some phytocannabinoids can interact directly with DOR, KOR and MOR. Moreover it was shown that increased activity of CB2 receptor leads to release of endogenous opioid peptides (e.g., β-endorphin) [41,42], which may partially explain the analgesic effect of cannabinoids. Moreover, CBD and THC are allosteric modulators of ligand binding to DOR and MOR [43].

The involvement of CBD in inflammation and redox balance modulation is in the effect of regulation of the calcium equilibrium in cells, which in turn plays a key role in cell proliferation processes, as well as in the secretion of pro-inflammatory cytokines [35]. Moreover, phytocannabinoids act at α2 adrenergic receptors and 5-HT_1A_ receptors, which in turn makes it possible to explain the antioxidant activity and the regulation of inflammatory processes by these compounds, even at a very low affinity to cannabinoid receptors [44,45]. 5-HT_1A_ receptors are activated by phytocannabinoids and capture reactive oxygen species, thus acting as a membrane antioxidant, protecting biological membranes from oxidative damage. The α2 adrenergic receptors, on the other hand, as a result of their activation by CBD, cause the release of adenosine (which exhibits anti-inflammatory properties) and reduce vascular cell adhesion molecule (VCAM-1) levels. These receptors can also bind with CB1 receptors, as a result of which the activity of both the receptors is altered [34]. Regardless of the direct action at membrane receptors, by inhibiting the activity of enzymes connected with the metabolism of fatty acids (cyclooxygenase 1/2), phytocannabinoids contribute to the reduction of lipid mediators’ generation, including eicosanoids, which reduce the activity of enzymes metabolizing endocannabinoids to fatty acids, including FAAH and MAGL [2,32], and participate in the regulation of the immune response [46]. The action both through receptors and through lipid mediators is conducive to the altering of redox balance and inflammation [47,48].

Considering the multidirectional metabolic activity in vitro, phytocannabinoids have been mainly studied so far as therapeutic agents in the treatment of neurological disorders [49], including the promising results of their use in the treatment of pain associated with multiple sclerosis and the alleviation of symptoms of Alzheimer’s or Parkinson’s disease [6,16], as well as diseases of the gastrointestinal tract such as Crohn’s disease or inflammatory bowel syndrome [17]. Moreover, it has been shown in the last decade that compounds from the group of phytocannabinoids have the ability to prevent the proliferation of cancer cells, which makes it possible to include them in the group of compounds with anticancer properties [18,19] against a broad range of cancers (including, e.g., breast, liver, pancreatic, colorectal, brain, lung cancer, or leukemia), with the effects observed in both in vitro and in vivo studies, as well as in clinical trials [19]. Additionally, there have been reports in recent years about the positive effects of phytocannabinoids on the skin and those of its cells that are characterized by altered metabolism as a result of solar radiation or the development of skin diseases [20].

## 5. Effect of Phytocannabinoids on Skin

The skin is an organ involved in communication between the human organism and the environment, and its main function is to protect the body against external physicochemical and biological factors, which are also the main etiological factors of skin diseases. The natural physical factor that constantly affects the skin cells is sunlight, which contains UV radiation that can disturb the metabolism of skin cells, but in a controlled way, is also used in the treatment of skin diseases [50,51]. As a consequence, research on the effectiveness of the pharmacological action of phytocannabinoids is related to the assessment of the independent impact of these compounds on skin cells, as well as the effects after UV irradiation (in vitro or ex vivo examinations) on skin cells, or during its direct exposure to the skin (in vivo studies) [52,53,54,55]. The skin is also exposed to chemical agents (e.g., disinfectants such as hydrogen peroxide) and pathogens (bacteria/viruses) [56,57]. Exposing the skin/skin cells to the above-mentioned physicochemical and biological factors usually leads to oxidative stress and inflammation in the skin cells, which may result in pathological changes and the development of skin diseases [58,59]. Consequently, the metabolic response of skin/skin cells to the above-mentioned factors, both potentially harmful and preventing such changes, is the subject of ongoing research. Taking into account that synthetic drugs usually generate side effects, more and more often, attempts are made to introduce preparations containing natural ingredients into pharmacotherapy, which usually cause fewer side effects. Therefore, given both the proven and potential pharmacological effects of phytocannabinoids, their usefulness in the topical treatment of skin diseases is increasingly being assessed [60,61,62]. 

### 5.1. Effect of Phytocannabinoids on the Metabolism of Healthy Skin Cells as Well as Healthy Skin Exposed to Physicochemical and Biological Factors—In Vitro Examinations

Keratinocytes and fibroblasts, which are the basic elements of the structure of the epidermis and dermis used in ex vivo and in vitro tests, are used for the initial assessment of metabolic changes resulting from the development of the disease at the skin level. Ex vivo tests utilize diseased skin cells isolated and treated with potentially therapeutic compounds immediately after collection, while in vitro tests are carried out on commercial cell lines (including immortal cells).

In vitro studies using keratinocytes from healthy donors showed that CBD cumulates in the cytosol of keratinocytes to a larger degree than in cell membranes [50]. Moreover CBD, the most studied phytocannabinoid, was reported to possess antioxidant and anti-bacterial properties [63]. Moreover CBD, the most studied phytocannabinoid, was reported to possess antioxidant and anti-bacterial properties [64]. It is believed that the antioxidant effect of CBD, under physiological conditions, is the result of CBD forming adducts with cytosolic (Keap1) and nuclear (Bach1) inhibitors of Nrf2, which promotes the activation of the Nrf2/ARE pathway, as a result of which the transcription factor Nrf2 enhances the transcription of cytoprotective proteins, including antioxidants [55]. At the same time, CBD, by inducing IκB kinase, contributes to the intensification of the degradation of the NFκB transcription factor, which reduces the pro-inflammatory response of cells [53]. However it was also indicated, that CBD, but stronger another phytocannabinoid—CBG effectively reduces reactive oxygen species level enhanced by UVA/B irradiation [52]. Consequently, phytocannabinoids antioxidant action is conducive to a reduction of UV-induced pro-oxidative stress and they protect against chemical and bacteria-induced inflammation (examined as pro-inflammatory cytokines such as TNFα and IL-6) in human dermal fibroblasts and normal human epidermal keratinocytes [52].

The consequence of antioxidant activities is the protective effect of phytocannabinoids, particularly CBD, in relation to macromolecular compounds—proteins and lipids, metabolically important cellular components. This results in a reduction in the level of oxidative modifications of protein and lipid structures [34]. It has been shown that decreasing phospholipid metabolism, both ROS-dependent and the enzymes-dependent (COXs and LOXs), reduces the formation of reactive products of lipid peroxidation (including unsaturated aldehydes such as 4-HNE), which, by forming adducts with proteins, change their structure and functionality [65] as well as lipid mediators from the group of eicosanoids and endocannabinoids, which also may modulate oxidative and inflammatory conditions [66]. Moreover it has been shown that CBD has a protective effect on the phospholipid membrane structures of keratinocytes of healthy people exposed to UVB radiation in vitro, preventing the upregulation of phosphatidylcholines and phosphatidylethanolamines [67]. In addition, preventing UV-intensified generation of lipid mediators, especially pro-inflammatory types (e.g., 4-HNE, PGE2) is conducive to reduced inflammation [47,63]. 

The levels of ROS and reactive phospholipid metabolites are diminished by CBD, which also reduces UV-induced changes in the structure, and thus the function, of proteins [54]. By reducing the expression of metalloproteinases, this phytocannabinoid protects intracellular matrix proteins from UV-induced pathological degradation [52,53]. In addition, it has been shown that the use of CBD after UV irradiation increases the expression of integrins that are necessary for the movement of collagen fibers necessary in the processes of cell growth regulation and wound healing [53]. Regardless of the proven cytoprotective effect of cannabidiol against keratinocytes treated with physical factors (UVA/B radiation) in vitro conditions, the protective effect in relation to the metabolism of skin cells has been demonstrated also in the case of causing stress by a chemical agents, such as hydrogen peroxide, used e.g., for skin disinfection [68]. 

### 5.2. Effect of Phytocannabinoids on the Metabolism of Healthy Skin Cells as Well as Healthy Skin Exposed to Physicochemical and Biological Factors–In Vivo Examinations

The protective effects of phytocannabinoids in in vitro conditions were confirmed by in vivo studies in which the skin of nude rats was exposed to UVA/UVB radiation and treated with CBD for 4 weeks, which confirmed both the antioxidant and anti-inflammatory effects of CBD on isolated skin cells [67,69,70]. It is suggested that this is due to the fact that CBD prevented the reduction of endocannabinoid levels under the influence of UV radiation, and consequently promoted the increase in the expression of cannabinoid receptors (CB1 and CB2) and TRPV1, which are involved in the regulation of ROS levels. It was also associated with reduced activity of cellular enzymes responsible for ROS generation and increased expression/activity of antioxidant components of glutathione- and thioredoxin-dependent antioxidant systems responsible for protection of lipids against ROS modifications, the decrease of which was observed after UV radiation on the skin of nude rats [70]. This resulted, among other effects, in limited reduction of the level of phosphatidylserine as a result of UV irradiation of the skin [71], which plays a key role in the regulation of oxidative stress, and thus the survival of skin cells. In addition, the consequences of applying CBD to the skin of rats were observed e.g., a reduction of oxidative modification of lipids [69,70]. The aforementioned multidirectional metabolic effects of cannabidiol applied to the skin during UV therapy clearly indicate that this phytocannabinoid, and therefore also other phytocannabinoids, can be considered as a factor protecting skin cells against the negative effects of environmental factors [68]. 

Confirmation of this direction of action of phytocannabinoids are also their antibacterial and antifungal properties, which prompts attempts to use them, among other cases, in the treatment of skin diseases such as psoriasis, atopic dermatitis, seborrheic dermatitis or acne [49]. Therefore, taking into account both the proven and potential pharmacological effects of phytocannabinoids in skin diseases, they are increasingly being tested for their usefulness in both topical and systemic applications [60,61,62]. It is also important due to the fact that natural substances/compounds have either no side effects or fewer side effects compared to comparable synthetic pharmacological preparations.

## 6. The Use of Phytocannabinoids in Counteracting Metabolic Changes Accompanying Skin Diseases

Despite the fact that the literature data clearly indicate that the cause of the pathological mechanisms of skin diseases, apart from inflammatory processes, is a disturbed balance between the level of reactive oxygen species (ROS) and the effectiveness of endogenous antioxidants, the precise mechanisms causing the development of skin dis-eases, and therefore also the methods of therapy, remain subject of research, particularly in vivo examinations [59,63,70,72,73,74,75]. As a consequence, effective pharmacotherapy of skin diseases usually involves compounds/preparations whose action is both antioxidative and anti-inflammatory, with little side effects [76]. Therefore, in recent years, research has been conducted to select and test natural compounds that would be therapeutically effective, but would not show harmful effects. The group of potential protective/therapeutic compounds in skin diseases includes phytocannabinoids, the potential of their metabolic actions being tested individually or in multicomponent combinations (Table 2). Plant extracts/oils containing mixtures of different compounds are evaluated [53,63,67,77]. 

### 6.1. Metabolic Changes in Psoriatic Skin Cells 

Among skin diseases, autoimmune skin diseases are relatively common dermatoses, which are characterized by significant diagnostic and therapeutic problems. However among the dermatoses, one of the most common is psoriasis, a chronic inflammatory skin disease of complex etiology [78], the incidence of which is constantly increasing and affects 2–4% of the world’s population, depending on the country, with about 1% affecting children and adolescents [79,80]. The development of psoriasis is associated with the action of environmental factors (bacterial or viral infections, trauma, stress) mainly on genetically predisposed individuals (strong correlation with some histocompatibility complexes, such as HLACw6 or HLACw7), which leads to the activation of immune cells [81,82,83], resulting in chronic inflammation within most skin cells and metabolic changes throughout the whole organism. Consequently, in addition to the changes typical for Psoriasis vulgaris (PsV) [84], 6–42% of patients with PsV develop Psoriatic arthritis (PsA), which is a progressive disease of the joints [85]. The characteristic clinical features of PsA include concomitant psoriatic skin lesions with accompanying pain, stiffness and swelling of the joints with limited mobility [86]. 

**Figure 2:**
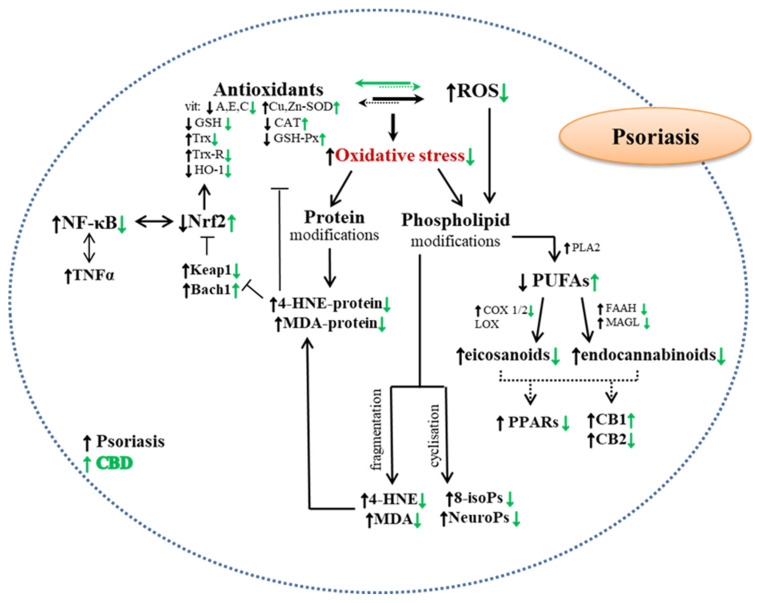
The effect of cannabidiol (CBD) on redox imbalance, inflammation and their metabolic consequences in the keratinocytes of patients with Psoriasis. Abbreviations: 4-HNE—4-hydroxynonenal; 8-isoPs—8-isoprostanes; Bach1—protein; CB1—cannabinoid receptor 1; CB2—cannabinoid receptor 2; COX—cyclooxygenase; FAAH—fatty acid amide hydrolase; Keap1—kelch-like ECH-associated protein 1; LOX—lipoxygenase; MAGL—monoacylglycerol lipase; MDA—malondialdehyde; NeuroPs—neuroprostanes; NFκB—nuclear factor kappa-B; Nrf2—nuclear factor erythroid 2; p65—nuclear factor NF-kappa-B p65 subunit; PLA2—phospholipase A2; PPARs—peroxisome; proliferator-activated receptors; PUFAs—polyunsaturated fatty acids; TNFα—tumor necrosis factor alpha.

In the classic form, i.e., psoriasis vulgaris, skin lesions intensify due to excessive keratinocytes proliferation (mainly caused by overproduction of IFNγ, IL-22 and IL-23) and epidermal accumulation [87]. However IFNγ stimulates skin cells to produce IL-1 and IL-23, which together with IL-6 participate in the activation of lymphocytes Th17 [88]. In keratinocytes and fibroblasts, this is accompanied by an increase in the activity of enzymes responsible for the production of ROS, and consequently enhanced level of ROS [63,89,90], as well as the deregulation of antioxidant capacity resulting from the reduced efficiency of the GSH-dependent system (GSH and GSH-Px), reduced CAT activity and the level of tocopherol, with a simultaneous increase in the efficiency of the Trx-dependent system (Trx and TrxR), which in turn leads to redox imbalance and oxidative stress generation [63,70] (Figure 2). Oxidative stress occurs both in the cells of the epidermis and the dermis of patients with psoriasis, which consequently promotes oxidative modifications of lipids and proteins [59,63,67,91]. Increased peroxidation of membrane phospholipids was found in keratinocytes and fibroblasts collected from psoriatic lesions, with increased levels of 4-HNE (in keratinocytes) and MDA and 8-isoprostanes (in keratinocytes and fibroblasts) [63,89,92], which resulted in decreased levels of phospholipid acids and changes in their location and functionality assessed on the basis of the membrane potential of cells [93]. In keratinocytes from psoriatic plaques, changes in the structure and functionality of proteins were also identified. They resulted mainly from the interactions between cysteine and histidine residues and reactive aldehyde formed in the process of lipid peroxidation (4-HNE) leading to the formation of 4-HNE-protein adducts [63,94]. It is believed that these modifications are particularly important for biological activity of Nrf2 transcription factor, because its cytosolic inhibitor—Keap1 is very susceptible to adduct with 4-HNE formation [95]. However Nrf2 is responsible for the biosynthesis of cytoprotective proteins, including antioxidant proteins, and is also involved in the regulation of the pro-inflammatory pathway at the level of pro-inflammatory cytokines, e.g., TNFα, which promotes the increase in the level of the NFκB transcription factor in keratinocytes and fibroblasts as well as may also enhance intercellular pro-inflammatory signaling [59,96,97]. In turn, in fibroblasts, increased oxidative stress favors oxidative modifications of proteins resulting from the formation of carbonyl groups in the protein structure [98]. This leads to increased MAPK activation (p38, ERK, JNK) and increased cell proliferation [89]. As a consequence, this process may increase the production of pro-inflammatory cytokines such as Il-6 and Il-8 by fibroblasts. This additionally intensifies the proliferation of keratinocytes, which is also favored by the increased expression of keratins: K6, K16 and K17 [99,100]. By intensifying protein modifications, oxidative stress in psoriasis also modifies the enzymatic metabolism of phospholipids, resulting in the formation of bioactive lipid mediators, mainly endocannabinoids and eicosanoids [63,77,101], which act on G protein-related receptors, including CB1/CB2, TRPV1 and PPARs (β and γ), as well as receptor-independent mechanisms, affect keratinocyte growth and differentiation, as observed in patients with psoriasis [63,102,103]. 

Despite the fact that the literature data clearly indicate that the cause of the pathological mechanisms of skin diseases, apart from inflammatory processes, is a disturbed balance between the level of reactive oxygen species (ROS) and the effectiveness of endogenous antioxidants, the precise mechanisms causing the development of skin diseases are still under investigation [59,63,70,73,74,75]. As a consequence, effective pharmacotherapy usually includes compounds/preparations with both antioxidant and anti-inflammatory effects, with minimal side effects [76]. Therefore, in recent years there have been studies aimed at finding/testing natural compounds that would be therapeutically effective, but would not show harmful effects. The group of potential protective/therapeutic compounds in skin diseases includes phytocannabinoids, the potential of their metabolic actions being tested individually or in multicomponent combinations (Table 2). Plant extracts/oils containing mixtures of different compounds are evaluated.

### 6.2. Influence of Phytocannabinoids on Redox Balance and Inflammation in Cells from Psoriatic Skin

One of the most studied (ex vivo and in vivo studies) non-psychoactive phytocannabinoids for possible topical psoriasis pharmacotherapy is CBD. Based on the literature data, it is difficult to clearly determine what membrane mechanisms, apart from the lipophilic nature of the compound, affect the penetration of skin cells by CBD. However, it has been shown that exposing keratinocytes, especially those irradiated with UVA/B radiation, and derived from the skin of patients with psoriasis to CBD, reduces the level of membrane transporters (ABC), which promotes the accumulation of CBD in membrane structures [63]. This fact suggests that CBD may limit the oxidative modifications of components, especially cell membranes, including phospholipids, and thus prevent the metabolic effects of peroxidation. This confirms the observed reduction in the levels of the lipid peroxidation product assessed as 4-HNE and the consequences of the reaction of this aldehyde with proteins in the form of 4-HNE-protein adducts. However, the presence of lower amounts of CBD in the cytosol of psoriatic cells does not mean that there is no effective metabolic effect at the intracellular level, nor does it mean that there is no possibility of at least partial prevention of the increased activation of pro-oxidative mechanisms in the keratinocytes of patients with psoriasis. This is visible in the reduced activity of the basic enzymes responsible for the generation of superoxide anion radicals (XO and NOX) [63], which results in their reduced level. This is additionally facilitated by the ability of CBD to chelate transition metal ions that catalyze the Fenton reaction [34], which diminishes the formation of hydroxyl radicals. Regardless of the reduction of pro-oxidative conditions, CBD also modulates the effectiveness of antioxidant system. This phytocannabinoid, by increasing the transcriptional activity of Nrf2 factor, has been shown to increase the activity of catalase and reduce the efficiency of the thioredoxin system, i.e., the cellular guardian of membrane phospholipids in the keratinocytes of patients with psoriasis [63]. The results of previous studies suggest that CBD reduces efficacy of GSH-dependent antioxidant system in keratinocytes of patients with psoriasis [63]. This is explained by the high affinity of CBD to GSH cysteine and GSH-Px selenocysteine [54], especially with more elevated levels of CBD in psoriasis cells. It was also suggested that the reactive metabolite of CBD - cannabidiol hydroxyquinone - reacts covalently with cysteine, creating adducts, e.g., with glutathione and cytochrome P450 3A11, thus inhibiting their biological activity [104]. CBD, on the other hand, supports the action glutathione peroxidase preventing the reduction of stannous ions necessary for the biological activity of this enzyme, the level of which is usually reduced in pathological conditions [105]. However, not all components of the antioxidant system are activated by CBD. The levels of both the main indicator of the antioxidant effectiveness of Nrf2, i.e., heme oxygenase, and the Nrf2 inhibitor Keap1, are reduced as a result of the application of CBD into cells derived from psoriatic skin of patients [55]. Nrf2 is also well known as an apoptosis inhibitor [106], which in psoriasis is not a beneficial phenomenon due to the fact that in this dermatosis hyperproliferation of keratinocytes is observed. In this disease other apoptosis regulating factors also plays important role in elimination of pathologically altered cells, such as antiapoptotic B-cell lymphoma 2 protein (Bcl2), proapoptotic Bcl2-associated X protein (Bax) [107]. It was shown that levels of phosphorylated form of Nrf2 (p-Nrf2) as well as Bax were decreased after CBD-treatment in keratinocytes obtained from psoriatic patients with simultaneously increased in Bcl2 level. On the other hand, in case of proapoptotic 15-deoxy-prostaglandin J2 (15-d-PGJ2) levels and antiapoptotic prostaglandin E2 (PGE2) levels in psoriatic keratinocytes an increase was observed [108].

In addition, it reduces the biosynthesis of TNFα, which may inhibit the external apoptosis pathway, which in turn may reduce the direct effect of TNFα on keratinocytes, including the activation of the pro-inflammatory NFκB pathway [109]. Consequently, this proves at least partly the protective activity of CBD with regard to cellular metabolism and shows multidirectional actions of this phytocannabinoid [59,67,77]. This phytocannabinoid actually protects phospholipids from modifications exacerbated by oxidative stress. However, in the case of proteins functioning in the cytosol, where the concentration of CBD is lower than in the structures of cell membranes, the protective effect is only partial (Figure 2).

**Table 2:** The influence of phytocannabinoids on metabolic changes in skin cells and on skin function disorders resulting from skin diseases.

Phyto- Cannabinoids	Phyto- Cannabinoid Concentration	Tested Cells/ Method of Application (Skin/Skin Cells)	Effects of Action	Ref.
**Psoriasis**				
in vivo examinations				
CBD	Ointment with CBD oil [retrospective studies]	Applied to the skin of patients with psoriasis	Increasing skin hydration, TEWL and elasticity level	[23]
ex vivo examinations				
CBD	4 μM	Keratinocytes from skin of psoriatic patients	Weakening the effects of UVB radiation Increased expression of Bcl2 apoptosis inhibitors, Changed the expression of apoptosis markers (p38 and caspase 8), Increased the level of 15-d-PGJ2 and PGE2, Induced MDM2 synthesis.	[77]
CBD	4 μM	Keratinocytes from skin of psoriatic patients	Reduced ROS level, Impact on changes in the level/activity of antioxidants, Reduced oxidative stress and its consequences [decrease in the level of 8-isoprostanes, 4-HNE and 4-HNE-protein adducts], Inhibited membrane transporters activity—MDR1, MRP, BCRP.	[63]
in vitro examinations				
CBG/CBN/Δ^9^-THC	100–200 mM	Human keratinocytes (HPV-16 E6/E7)	Inhibition of keratinocytes proliferation	[110]
**Acne**				
in vitro examinations				
CBC	0–10 μM	Human sebocytes (SZ95)	Anti-inflammatory effect, reduction of natural and induced AA synthesis,	[111]
THCV	0.1–10 μM	Human sebocytes (SZ95)	Anti-inflammatory effect, Inhibition of proliferation	[111]
CBGV	0.1–10 μM	Human sebocytes (SZ95)	Anti-inflammatory effect, Increased synthesis of sebaceous lipids,	[111]
**Seborrhea**				
in vitro examinations				
CBD	1–10 μM	Human sebocytes (SZ95)	*Lipostatic effect* Inhibited lipogenesis, counteracting acne-inducing agents, Increased expression of ARHGAP9. *Anti-proliferative action* Increase of intracellular Ca^2+^ levels through TRPV4 agonism, Increased TRPV4 activation in initiating antiproliferative signaling cascade, Decreased level of MKI67. *Anti-inflammatory effect* Changed expression of the antimicrobial peptide LL-37 cathelicidin and TRIB3, Inhibition of the p65-NFκB pathway, RTeduced MKI67 marker expression.	[112]
**Allergic contact dermatitis**				
in vitro examinations				
CBC	1–20 μM	Keratinocytes (HaCaT) stimulated with poly-(I:C)	Reduction of MCP-2, IL-6 and IL-8 levels	[113]
**Atopic dermatitis**				
in vivo examinations				
CBD	Ointment with CBD oil [retrospective studies]	Applied to the skin of patients	Reduced secretion of excessive skin sebum Improved skin elasticity and hydration	[23]

Abbreviations: 15-d-PGJ2—15-deoxy-prostaglandin J2; 4-HNE—4-hydroxynonenal; AA—arachidonic acid; Bcl-2—B-cell lymphoma 2; BCRP—breast cancer resistance protein; CB1—cannabinoid receptor 1; CB2—cannabinoid receptor 2; CBC—cannabichromene; CBD—cannabidiol; CBG—cannabigerol; CBGV—cannabigerovarin; CBN—cannabinol; IFNγ—Interferon gamma; IL-6—Interleukin 6; IL-8—Interleukin 8; MCP-1—monocyte chemoattractant protein 1; MCP-2—monocyte chemoattractant protein 2; MDM2—E3 ubiquitin-protein ligase Mdm2; MDR1—multidrug resistance protein 1; MRP—multidrug resistance protein; PGE2—prostaglandin E2; poly-(I: C)—polyinosinic-polycytidylic acid; p-p38—phospho-Tyr182; ROS—reactive oxygen species; THC—tetrahydrocannabinol; THCV—tetrahydrocannabivarin; UV—ultraviolet irradiation.

The antioxidant effect of CBD on membrane phospholipids leads to a reduction in changes in pro-oxidative activity resulting from the development of psoriasis, which is clearly indicated by the reduced level of oxidative modification products of these compounds, assessed as 8-isoprostanes and low molecular weight aldehydes (4-HNE and MDA) present in the keratinocytes and fibroblasts of patients with psoriasis [63,92,93]. As a consequence, this is accompanied by a decrease in the level of 4-HNE-protein adducts, which suggests that 4-HNE changes the structure of proteins to a lesser extent, including metabolically active ones, which may affect signal transmission (e.g., by stimulating kinase), thus acting beneficially from a metabolic point of view [63]. A lower degree of protein modification in the skin cells of patients with psoriasis treated with CBD also has the effect of reducing the intensity of the enzymatic metabolism of phospholipids, which can be seen in the reduced level of their metabolites, such as endocannabinoids and eicosanoids [63,77]. However, other data show that CBD, as a phytocannabinoid, inhibits the cellular uptake and catabolism of the endocannabinoid (AEA), competing with AEA for binding with fatty acid binding proteins (FABP) responsible for the transport of hydrophobic compounds into the cytosol [114]. It has been noted that CDB does not affect the activity of these enzymes that break down endocannabinoids in the keratinocytes of patients with psoriasis, but reduces their levels in cases of previous UV irradiation [63]. As a consequence, a change in the expression of membrane receptors such as CB1/2, TRPV and PPAR is observed, which leads to a decrease in the level of ROS and pro-inflammatory cytokines and cell proliferation [63]. Moreover, by reducing the activity of enzymes involved in PUFA metabolism, CBD also reduces the production of eicosanoids, including pro- and anti-apoptotic prostaglandins (15-d-PGJ2 and PGE2) in the keratinocytes of patients with psoriasis [77], with the effect of CBD on the level of 15 -d-PGJ2 may possibly lead to the activation of the extrinsic apoptotic pathway, which may indicate its use in a more selective treatment of psoriasis [77]. 

The effect of the intense CBD activity at the level of cell membranes, however, is the considerable reduction of the level of phosphatidylcholines, phosphatidylserines, and most classes of phosphatidylethanolamines in keratinocytes isolated from psoriasis patients [92], at a simultaneous increase of the level of sphingomyelins [67]. At the same time, the tendency to upregulate is observed for most classes of ceramides both in keratinocytes and fibroblasts of psoriasis patients, which may be a significant response to the inflammatory processes observed in psoriatic skin [67]. This is the more important aspect as ceramides, which are products of metabolism of sphingomyelins, are regarded as the most important lipid metabolites connected, above all, with the permeability of cell membranes [115]. It has been shown that CBD also has an impact on the increase in the level of aquaporin-3 (AQP3) (at the level of mRNA and protein), which plays an important role in water retention in the skin, in psoriasis patients as well [116], thus improving its hydration. This is also confirmed in in vivo studies, which show an increased water content in the skin of mice subjected to the action of CBD [117]. 

Regardless of the presented data concerning the influence of CBD on psoriatic skin cells, there is also other literature data that indicates that both CBD and other phytocannabinoids (THC, CBN, and CBG) inhibit the proliferation of keratinocytes (intensified in psoriasis) in a concentration-dependent manner, as shown in in vitro studies with the use of hyperproliferating human keratinocyte cells HPV-16 E6/E7 [110]. 

Irrespective of local metabolic changes in skin cells, the development of psoriasis is connected with the consequences of oxidative stress and inflammations that affect the whole body and lead to metabolic changes observed systematically in the blood [94,108,118]. There is no literature data, however, on the direct influence of phytocannabinoids on metabolic changes in the blood caused by psoriasis; it is therefore difficult to predict what changes would be observed in the area of blood or blood cells metabolism as a result of a therapy that uses phytocannabinoids. However, studies performed on rats with CBD chronically applied to skin show that the phytocannabinoid is carried with blood to the inside of the organism, where it may display a therapeutic effect [119].

Regardless of the data and suggestions concerning natural phytocannabinoids presented above, it may be suggested that apart from the discussed compounds, synthetic derivatives of cannabinoids may also exhibit positive effects in the treatment of psoriasis, including JWH-133, which displays antiangiogenic and anti-inflammatory properties, and inhibits the production of several angiogenic growth factors (e.g., HIF-1α, VEGF, MMP, and bFGF) and cytokines (e.g., IL-8 and IL-17). This indicates that they may have therapeutic properties in inflammations and angiogenesis, which are effects of psoriasis [120].

However, despite this extensive basic research, including on skin cells, there is a very limited amount of clinical trials indicated on the beneficial effects of phytocannabinoids on the metabolic changes that accompany the development of psoriasis and other skin diseases.

## 7. Clinical Studies on the Effect of Phytocannabinoids on Skin Diseases

Both the individual components, mainly phytocannabinoids, and *Cannabis sativa* extracts have been intensively studied in recent years in terms of their biological effects, especially those that protect healthy skin and heal the affected skin (Table 3) [52,109,121,122,123,124]. However, despite the demonstrated wide range of biological properties, there is very little evidence based on clinical trials indicating the possibility of using specific preparations containing phytocannabinoids in medical practice. The best-studied non-psychoactive phytocannabinoid is cannabidiol (CBD), which, in addition to its antioxidant and anti-inflammatory properties, has antibacterial, anti-acne and anti-aging effects when applied topically to the skin [125]. Clinical studies conducted on relatively small groups of volunteers have shown a reduction in erythema and sebum secretion under the influence of CBD, which can be used in the case of dry skin and acne [112]. This has been confirmed by phase I clinical trials showing that a patented high-purity CBD preparation (BTX 1503) obtained through the use of yeast fermentation technology reduces acne lesions [122]. Notwithstanding the similarities of various phytocannabinoids in their biological and biomedical effects, in addition to the extensively researched effects of CBD, including clinical effects, recent years have brought new detailed data on other components of *Cannabis sativa*, including THC and CBG. Oil containing CBD and THC has been shown to have the ability to accelerate wound healing and reduce blistering in people with Epidermolysis bullosa (EB) - a genetic skin disease characterized by the formation of blisters on the surface of the skin [123]. The use of oil contributes to the reduction of analgesic pharmacotherapy.

Despite Despite the similarities of various phytocannabinoids in their biological and biomedical effects, in addition to the extensively researched effects of CBD, recent years have brought new detailed data on other components of *Cannabis sativa*, including THC and CBG. CBG, with a broad spectrum of biological effects on skin cells in vitro, including antioxidant, anti-inflammatory, anti-acne and anti-aging properties, has also been shown in clinical trials to be both safe and effective in maintaining skin health, including reducing redness [52,121]. Therefore, taking into account the demonstrated protective effect observed in both in vitro and in vivo studies, CBG has become a new candidate for dermatological use. In addition, both CBG and CBD have been shown to have potent antimicrobial activity against bacteria such as *Cutibacterium acnes*, *Staphylococcus aureus* and methicillin-resistant *S. aureus* (MRSA) [127]. Since S. aureus is a key factor in the pathogenesis of atopic dermatitis [128], it may be highly possible that CBG may also be effective in this dermatological condition. Given this broad profile of action, it has been hypothesized that CBD, CBG and other phytocannabinoids may be effective in treating acne-prone skin. CBG enhanced potency over CBD in reducing inflammation caused by *C. acnes* has been shown to make CBG an attractive candidate for acne treatment used separately or in combination with other cannabinoids. 

The use of oil contributes to the reduction of analgesic pharmacotherapy. Moreover, recent years have brought new data from clinical trials on other components of *Cannabis sativa*, including THC and CBG. Oil containing CBD and THC has been shown to have the ability to accelerate wound healing and reduce blistering in people with Epidermolysis bullosa—a genetic skin disease characterized by blistering of the skin surface, with the use of the oil helping to reduce the effect of pain medication [123]. 

Despite the still small number of clinical trials, the possibility of therapeutic use of phytocannabinoids in skin diseases makes their participation in skin preparations has become more and more common in recent years [52,129]. This is also evidenced by patent applications and granted patents for the use of phytocannabinoids in medicinal preparations, e.g., in the pharmacotherapy of psoriasis by topical application of a composition containing cannabinoids, in particular CBD and CBG at a concentration of 3–20% by weight [24]. 

## 8. Conclusions

The above presented data as well as legislative changes regarding the use of preparations/compounds derived from *Cannabis sativa* indicate that there is a real future for the use of hemp ingredients in the treatment of skin diseases, including psoriasis, but only on the condition of conducting clinical trials on a large group of patients, taking into account both the two clinical manifestation of psoriasis (psoriasis vulgaris and psoriatic arthritis), as well as various degrees of severity of the disease and coexisting diseases, especially of the skin. In addition, ultimately, due to the possibility of different disease etiologies the research should concern children, young people and adults independently. Moreover, considering the influence of UV radiation on the course of the disease, clinical trials should also take into account this aspect of therapy. Only the multidirectional approach applied in clinical trials will actually show not only the possibilities, but also the limitations of the use of various preparations/compounds derived from *Cannabis sativa*.

The appearance of synthetic equivalents of phytocannabinoids (including high-purity compounds) in research also indicates an active approach of the global scientific community to the problem of pharmacotherapy of skin diseases, including psoriasis. It can therefore be hoped that there is also a chance to generate synthetic derivatives of phytocannabinoids modified for maximum pharmacological effectiveness and minimize side effects.

## Figures and Tables

**Table 1:** The affinity of phytocannabinoids for G protein-coupled receptors [30,34,38,39].

Receptors	Phytocannabinoids					
	CBG	CBC	CBD	Δ^9^-THC	Δ^8^-THC	CBN
CB1	wa	pa	wa	a	pa	wa
CB2	a	pa	want	pa	pa	a/ra
GPR18	NDF	NDF	ant	a	NDF	NDF
GPR55	NDF	NDF	ant	wa	NDF	NDF
TRPV1	a	a	a	a	NDF	a
TRPV2	a	a	a	a	NDF	a
TRPM8	ant	ant	ant	ant	NDF	ant
TRPA1	a	a	a	a	NDF	a
5-HT_1A_	ant	NDF	a	ant	NDF	NDF
α_2_-AR	a	NDF	a	NDF	NDF	NDF
PPARγ	a	NDF	a	a	NDF	NDF

Abbreviations: CB1—cannabinoid receptor 1; CB2—cannabinoid receptor 2; GPR18—G protein-coupled receptor 18; GPR55—G protein-coupled receptor 55; TRPV1—transient receptor potential channel vanilloid type 1; TRPV2—transient receptor potential channel vanilloid type 2; TRPM8—transient receptor potential channel melastatin type 8; TRPA1—transient receptor potential channel ankyrin type 1; 5-HT_1A_—human serotonin receptor; α2-AR—adenosine A_2A_ receptors; PPARγ—peroxisome proliferator activated receptors gamma. a–agonist, ant–antagonist, w–weak interaction, p-partial interaction, r-reversed interaction, NDF—no data found. The table does not include cannabinodiol (CBND), cannabielsoin (CBE), cannabicyclol (CBL) and cannabitriol (CBT), for which no literature data was found.

**Table 3:** The use of phytocannabinoids in clinical trials.

Cannabis sativa/Phyto- cannabinoids	Test Products/ Time of Application	Method of Application	Skin Problems	Total Participants	The Effects of the Preparation	Suggestion for Treatment	Ref.
CBD + CBG oil	3–20% 3 and 15% CBD/CBG oil (2:1—ratio of compounds) twice a day for 6 weeks	Topically on psoriatic lesions on the arms	Psoriasis vulgaris	2 patients	Reduction of psoriatic lession	Psoriasis vulgaris	[24]
CBD	5% CBD solution twice a day for 84 days	Topically on face with acne changes	Acne vulgaris	368 patients	Reduction of acne lession	Acne vulgaris	[126]
Cannabis seeds extract	3% extract cream [12 weeks extract + base and base twice a day]	Topically (on the cheeks)	Sebum and erythema (human cheek)	11 healthy volunteers	Reduction of skin sebum and erythema	Acne vulgaris, seborrhea, papules	[121]
Synthetic CBD (BTX1503) PermetrexTM Patent	5% CBD [14 and 28 days -twice a day]	Topically	Acne vulgaris	20 healthy volunteers + 23 people with moderate to severe acne	Safe and well tolerated; anti-acne effect	Acne vulgaris	[122]
CBM oil (CBD + THC)	CBD (20 mg/mL) + THC (13 mg/mL) [from several months up to 2 years]	Sublingually	Epidermolysis bullosa	3 patients with EB; without control	Reducing pain/itching and taking painkillers	Epidermolysis bullosa	[123]
CBG	0,1% CBG serum/placebo [2-weeks after irritation]	Topically on the skin after sodium lauryl sulfate (SLS)-induced irritation	Skin irritation	20 healthy volunteers	Reduced inflammation, Redness, improved the skin barrier (TEWL)	Skin problems after inflammatory inducers (physical/chemical)	[52]
CBD + PEA	1% CBD + PEA (gel, balm); (0,1–10% CBD); hemp seed oil- without CBD; 0.9% NaCl and 0.1% w/v SLS as control [21-days cumulative test]	Topically (skin of the back)	Normal skin	20 healthy volunteers with exclusion criteria	No increase in skin irritation or sensitization	Possibility: long-term use of medicinal/cosmetic preparations containing CBD and PEA	[124]
CBD + PEA	balm (0.1% CBD and PEA) cream (hemp seed oil, no CBD) + UVA irradiation [24 h after]	Topically (skin of the back)	Skin irradiated	22 healthy volunteers with exclusion criteria	No differences in phototoxicity vs. negative control	Possibility: using preparations on skin exposed to solar radiation	[124]

## Supplementary Materials

The following supporting information can be downloaded at: https://www.mdpi.com/article/10.3390/molecules28031192/s1, Figure S1: Flow diagram of literature search.
