# Supplementary material for: Phytocannabinoids in the Pharmacotherapy of Psoriasis
Source: Molecules. 2023 Jan 25;28(3):1192. doi: 10.3390/molecules28031192 (PMC9920113; doi:10.3390/molecules28031192)
Supplement: Supplementary file 1 [file molecules-28-01192-s001.zip › molecules-2147796-supplementary.pdf]

molecules

MDPI

Supplementary Material

Phytocannabinoids in the Pharmacotherapy of Psoriasis

Adam Wroński [1], Iwona Jarocka-Karpowicz [2], Anna Stasiewicz [2] and Elżbieta Skrzydlewska [2,*]

[1] Dermatological Specialized Center "DERMAL" NZOZ in Bialystok, 15-453 Bialystok, Poland
[2] Department of Analytical Chemistry, Medical University of Bialystok, Mickiewicza 2D, 15-222 Bialystok, Poland
* Correspondence: elzbieta.skrzydlewska@umb.edu.pl; Tel.: +48-857-485-882

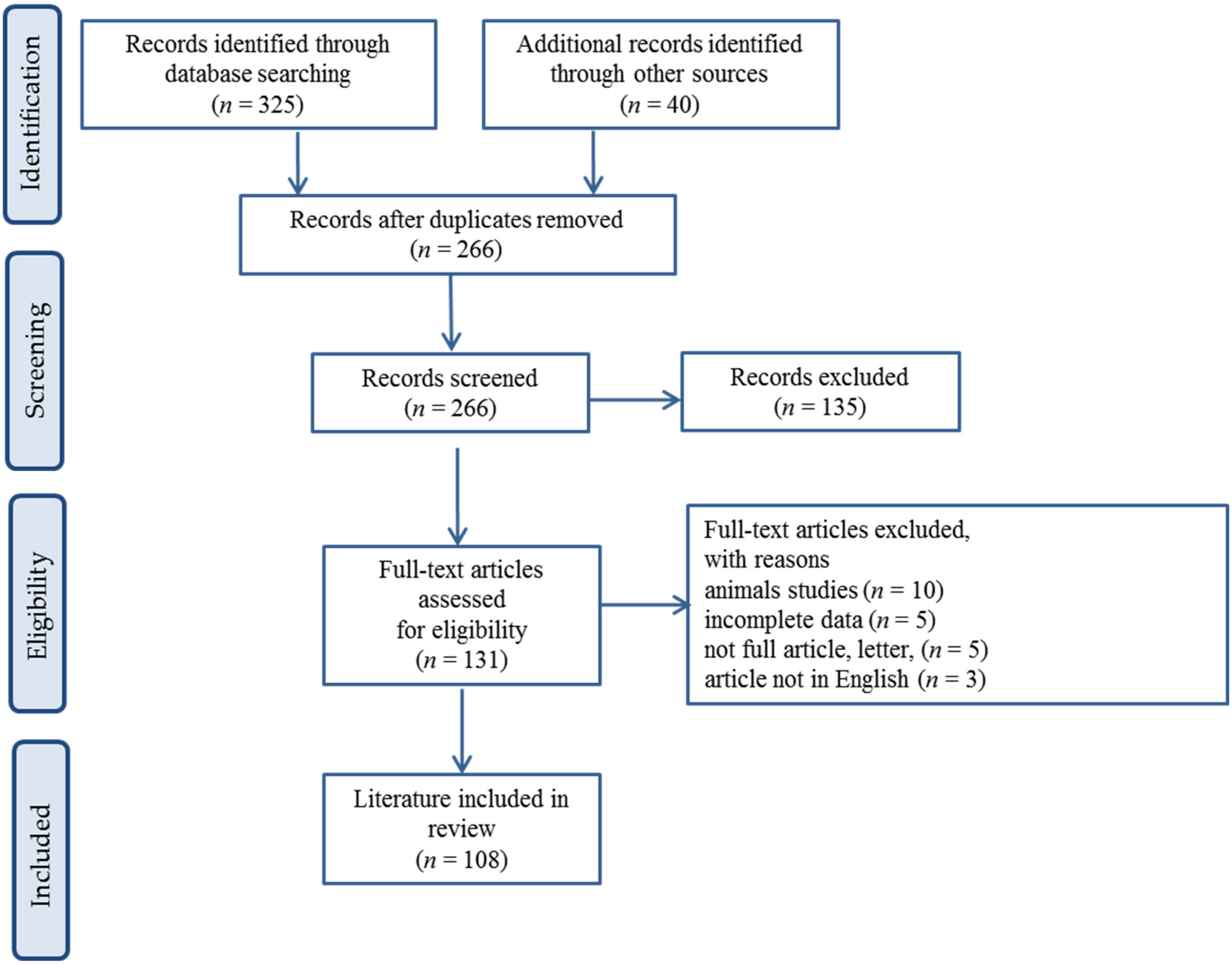

Figure 1. Flow diagram of literature search.
